# A standardized rat model of maxillary anterior periodontal soft tissue defect for the evaluation of soft tissue graft materials

**DOI:** 10.3389/fbioe.2026.1802902

**Published:** 2026-04-20

**Authors:** Ruoxuan Huang, Chunhsin Hsu, Shoucheng Chen, Junming Feng, Zetao Chen, Runheng Liu, Zhuofan Chen

**Affiliations:** Hospital of Stomatology, Guanghua School of Stomatology, Sun Yat-sen University and Guangdong Provincial Key Laboratory of Stomatology, Guangzhou, China

**Keywords:** angiogenesis, animal model, biomaterials evaluation platform, immune response, periodontal soft tissue regeneration

## Abstract

**Background:**

Functional reconstruction of periodontal soft tissue is a major focus in both clinical and basic research within the field of dentistry. However, standardized small-animal models that accurately recapitulate clinical periodontal soft tissue defect remain limited, hindering the development of the graft materials. This study aimed to establish a standardized, minimally invasive and reproducible rat model of periodontal soft tissue defect for systematic *in vivo* assessment of soft tissue regeneration.

**Methods:**

The anatomical characteristics of the maxillary and mandibular anterior labial regions and the hard palate in rats were comprehensively assessed to identify optimal recipient and donor sites. Based on these findings, a standardized 4 × 2 mm soft tissue defect was created in the maxillary anterior labial region and reconstructed using autograft, xenograft or left untreated as a control. The healing process was evaluated through sequential clinical observation, H&E staining, Masson’s trichrome staining and immunohistochemical analyses.

**Results:**

The results demonstrated that this model enabled consistent defect creation with low complication rates and high reproducibility. Distinct yet clinically relevant healing patterns were observed among different treatment groups, closely resembling those reported in human periodontal soft tissue reconstruction. The model allowed dynamic evaluation of the quality of healing, tissue integration, materials degradation, angiogenesis and immune response of periodontal soft tissue regeneration.

**Discussion:**

This model provides a robust and translationally relevant *in vivo* platform for the mechanistic assessment of soft tissue graft materials. It facilitates systematic investigation of biological responses during soft tissue regeneration and supports early-stage screening and optimization prior to large-animal studies and clinical translation.

## 1 Introduction

Sufficient and healthy periodontal soft tissue not only provides a protective barrier against microbial invasion and mechanical trauma for teeth and dental implants, but also plays a crucial role in maintaining periodontal health, promoting soft and hard tissue healing, and achieving satisfactory aesthetic outcomes (Ríos-Osorio et al., 2025; Cho et al., 2021). In recent years, the functional reconstruction of periodontal soft tissues has emerged as a major focus of both clinical practice and basic research in dentistry (Liang et al., 2020). Without timely and effective treatment, defects in periodontal soft tissue may lead to gingival recession, loss of periodontal attachment, and even a compromise of the long-term stability of dental implants and overall oral health (Jensen et al., 2023; Toledano et al., 2020; Li et al., 2026). Hence, the development of novel soft tissue graft materials and the investigation of their *in vivo* biological effects are of considerable importance for advancing periodontal regenerative therapies.

Currently, periodontal soft tissue reconstruction primarily relies on autologous free tissue grafts (such as free gingival grafts and connective tissue grafts) and soft tissue substitutes (such as xenogenic collagen matrices and bioengineered scaffolds) (Li et al., 2026). Although autologous grafts provide predictable and well-documented clinical outcomes, their application is constrained by donor-site morbidity, increased surgical trauma, and limited tissue availability. In contrast, soft tissue substitutes have attracted growing interest due to their minimal invasive nature and abundant sources (Cho et al., 2021; Liang et al., 2020). Nevertheless, existing substitutes continue to face challenges related to biocompatibility, tissue integration, degradation kinetics and the promotion of neovascularization, and their long-term clinical performance remains to be improved (Li et al., 2026; Ríos-Osorio et al., 2025). Therefore, comprehensive *in vivo* evaluation and mechanistic investigations are essential for advancing these materials toward successful clinical translation.

Animal models are indispensable platforms for evaluating the *in vivo* effects and underlying mechanisms of soft tissue graft materials. Large animals (such as monkeys, dogs, and pigs) serve as ideal preclinical models because their oral soft and hard tissue anatomy closely resembles that of humans. However, the high economic cost, ethical concerns, surgical complexity, and lengthy experimental cycles associated with large-animal studies limit their use in early-stage material screening and dynamic, multi-time-point investigations (Domínguez-Oliva et al., 2023). Rats, as small-animal models, offer advantages including well-characterized genetics, extensive molecular biology toolkits, and low maintenance and experimental costs, making them widely used for the early biological evaluation of materials (Liu et al., 2019; Liu et al., 2018). Nevertheless, most existing rat models for soft tissue regeneration are based on skin wound healing, which fundamentally differs from oral mucosa in anatomy, blood supply, and microenvironment. Therefore, these models cannot accurately recapitulate the healing process of oral soft tissues (Toma et al., 2021). Although some studies have established rat palatal mucosal defect models to better mimic the oral environment, the complex palatal anatomy, rich vascularization, and high risk of surgical complications (including hemorrhage, nasal cavity injury, and material dislodgement) still limit their application as precise periodontal soft tissue defect models (Chaushu et al., 2020; Chen et al., 2023).

To date, standardized small-animal models that accurately recapitulate clinical periodontal soft tissue defects remain lacking. The main challenges include: (1) establishing a standardized and reproducible protocol for defect creation and material fixation within the confined intraoral space of rats; (2) Ensuring sufficient blood supply for periodontal soft tissue healing; and (3) effectively evaluating histological and molecular changes across multiple time points. In this context, our study aims to systematically characterize the anatomical characteristics of maxillary and mandibular anterior labial regions and hard palate in rats, identify optimal recipient and donor sites, and optimize surgical procedures to establish a standardized, minimally invasive, reproducible, and low-complication rat model of periodontal soft tissue defect. This model enables systematical comparison of histological healing, material degradation, neovascularization, and immune response associated with autografts and soft tissue substitutes during defect repair, thereby providing a theoretical and experimental basis for subsequent mechanistic investigations and clinical translation of novel soft tissue graft materials.

## 2 Materials and methods

### 2.1 Animals

**FIGURE 1:**
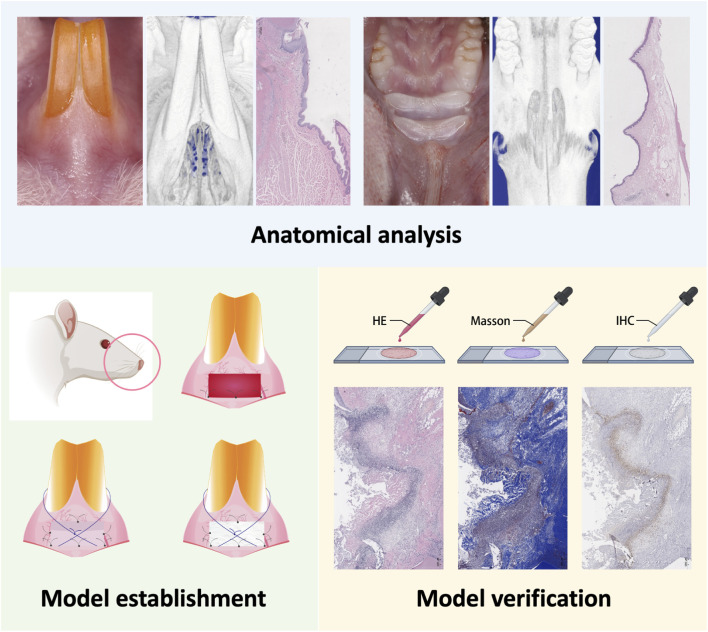
The experimental procedures of this study.

All animal procedures were approved by the Institutional Animal Care and Use Committee, Sun Yat-Sen University and complied with national and institutional regulations on animal welfare (Approval No. SYSU-IACUC-2022-000722). Twenty-seven healthy male Sprague-Dawley rats (14 weeks old, 450–550 g) were used in this study. The entire experimental procedures are shown in Figure 1.

### 2.2 Anatomical structure analysis

Rats were euthanized and the maxilla and mandible were harvested. Samples were fixed in 4% paraformaldehyde and scanned using micro-CT (Venus®, China) with a voltage of 70 kV, a current of 114 μA, and a spatial resolution of 10 μm. The acquired data were reconstructed and analyzed using SimPlant software (MaterialiseDental, Belgium) to evaluate the anatomical structures of the maxillary and mandibular anterior regions and the hard palate, with particular attention to mucosal thickness, bone morphology, and the distribution of blood vessels. After micro-CT scanning, the specimens were decalcified in 10% EDTA at room temperature until sufficiently softened. Tissues were dehydrated, embedded in paraffin, and sectioned at 4 μm thickness. Hematoxylin and eosin (H&E) staining was performed to observe the histological structure of the soft and hard tissues, including the thickness and composition of the mucosa and underlying connective tissue, as well as the presence and distribution of muscular and vascular tissues.

### 2.3 Model establishment

#### 2.3.1 Grouping

**FIGURE 2:**
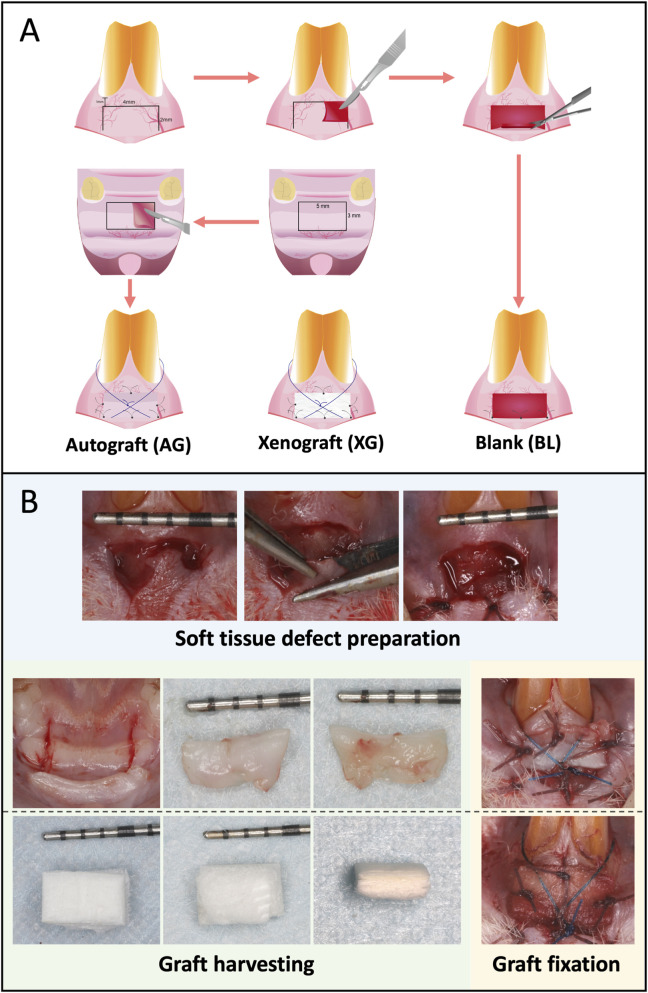
Grouping **(A)** and stepwise procedure for the establishment of the rat intraoral soft tissue defect model **(B)**. Soft tissue defect preparation: Creation of standardized soft tissue defects in the maxillary anterior labial gingiva. Graft harvesting: Harvesting of autologous palatal mucosal grafts and preparation of soft tissue substitute materials, with representative images showing graft dimensions. Graft fixation: Placement and fixation of grafts onto the defect site using interrupted sutures. The ruler provides scale for defect and graft size.

Based on the anatomical and histological analyses, the labial site of the maxillary incisors was selected as the recipient site for soft tissue defect creation, and the palatal mucosa anterior to the first molar was chosen as the donor site for autograft harvesting. A total of 27 rats were randomly divided into three groups and evaluated at three time points (n = 3 per group at each time point). Autograft group (AG): Soft tissue defects were repaired with autologous grafts harvested from the hard palate. Xenograft group (XG): Defects were repaired using a xenogenic collagen matrix (Mucograft®, Geistlich Pharma, Switzerland). Blank group (BL): Defects were left untreated as negative controls (Figure 2A).

#### 2.3.2 Surgical procedures

The surgical procedures were performed by a single operator with more than 5 years of experience in mucogingival surgery to avoid inter-operator variability. Prior to the formal experiment, three surgical procedures were performed for each group according to the following protocol, allowing the operator to become proficient and ensuring procedural consistency across animals. Rats were anesthetized with intramuscular injection of sodium pentobarbital (0.1 mL/100 g). A horizontal incision (4 mm in length) was made 1 mm apical to the gingival margin of the maxillary incisor, followed by two vertical incisions (1.5–2 mm in length). The defect size was kept consistent by measuring with a periodontal probe. A partial-thickness flap was elevated with a 15C scalpel, and the periosteum was preserved intact. The flap was removed using scissors. The apical mucosal margin was secured to the periosteum with three vertical-horizontal mattress sutures, creating a 4 × 2 mm standardized soft tissue defect. Autograft harvesting: A horizontal incision (5 mm in length) was made along the line connecting the mesial aspects of the bilateral first molars on the hard palate, with two vertical incisions (3 mm each) at both ends. A partial-thickness flap was elevated, and the submucosal tissue (including adipose and glandular tissue) was removed using scissors. The autograft (AG) was trimmed to 4 × 2 mm and transplanted to the defect site. Xenograft (XG) preparation: A xenogenic collagen matrix was trimmed to 4 × 2 mm and placed over the defect. AG or XG was secured to the defect with three interrupted 6-0 absorbable polyglycolic acid sutures and one additional cross suture using 6-0 non-absorbable polypropylene to ensure close adaptation to the wound bed. For the blank (BL) group, no graft was applied (Figure 2B). Postoperatively, rats received intramuscular penicillin (40,000 U) and analgesia (0.1 mL). Soft diet was provided until sacrifice.

### 2.4 Model verification

The healing process was monitored and photographed at 3, 7, 10, and 14 days postoperatively. Signs of bleeding, infection, graft loss, and changes in wound appearance were recorded. At 3, 7, and 14 days after surgery, rats were euthanized, and tissue samples (including maxillary incisors, gingiva, and alveolar bone) were harvested and fixed in 4% paraformaldehyde. After decalcification and embedding, H&E staining and Masson’s trichrome were performed following the protocol provided by the manufacturer. Immunohistochemical staining was performed for rabbit anti-CD31 (Abcam, UK), rabbit anti-CD68 (Abcam, UK), rabbit anti-iNOS (Abcam, UK) and rabbit anti-CD206 (Abcam, UK) (1:2000, 1:100, 1:500, 1:4000, respectively). Histological assessment included evaluation of graft integration, epithelialization, collagen remodeling, vascularization, and inflammatory cell infiltration.

### 2.5 Statistical analysis

Quantitative data were presented as mean ± standard deviation (SD), and one section per animal was analyzed. For blood vessels quantification, two random fields within the region of interest were selected, vessels were manually counted and averaged, and vessel density (vessels/cm^2^) was calculated. For macrophage markers quantification, five random fields were selected, positive cells were segmented based on OD values, and density was calculated by IOD/area using Image-Pro Plus software. Statistical analysis was performed using SPSS (version 22.0, IBM, USA). One-way analysis of variance (ANOVA) was used, followed by Bonferroni test. A *p*-value less than 0.05 was considered statistically significant.

## 3 Results

### 3.1 Anatomical analysis

**FIGURE 3:**
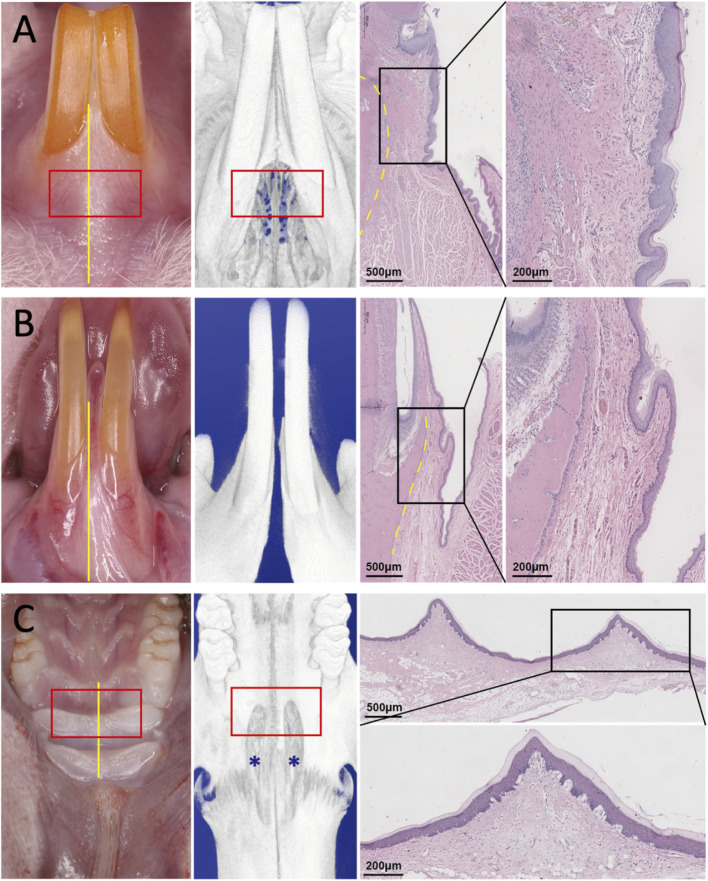
Representative images of gross view, corresponding micro-CT, and histological sections showing the anatomical structure of different intraoral regions in the rat. **(A)** Labial side of the maxillary incisors. **(B)** Labial side of the mandibular incisors. **(C)** Hard palate. The histological images show both low- and high-magnification views of the selected area. Scale bars are indicated in each panel. Blue asterisks indicate the sinus cavity. Yellow lines indicate the location of the slices. Dotted lines indicate the outline of alveolar bone.

Comprehensive histological and micro-CT analyses were performed to evaluate potential sites for soft tissue defect creation. Both the labial aspects of the maxillary and mandibular incisors demonstrated a distinguishable band of attached gingiva. Histologically, the labial mucosa consisted of a stratified squamous epithelium overlaying a subepithelial connective tissue, with muscular tissue present near the vestibular sulcus. Notably, the maxillary labial mucosa was significantly thicker than its mandibular counterpart. Based on these findings, the labial site of the maxillary incisors was identified as the optimal site for defect creation due to the following advantages: (1) The superior mucosal thickness enables more precise and safer surgical manipulation, including incision, flap elevation, and suturing, thereby minimizing the risk of laceration. (2) The thicker mucosa ensures a robust blood supply from the surrounding tissues, which is critical for graft integration and healing. (3) The anatomical configuration of the maxillary incisor region and its adjacent hard tissue closely resembles that of the human periodontal tissue, enhancing the translational relevance of the model (Figures 3A,B).

Examination of the hard palate revealed multiple parallel palatal rugae. Histological analysis demonstrated a gradual decrease in mucosal thickness from the anterior to the posterior palate. Micro-CT imaging further showed that, anterior to the maxillary molar, a sinus cavity was present and communicated with the submucosal tissue. Our previous study has revealed a dense vascular network in this sinus cavity, with major blood vessels running bilaterally along the posterior aspect of the hard palate (Liu et al., 2024). The palatal mucosa anterior to the maxillary first molar was selected as the ideal donor site for autograft harvesting because: (1) This region provides sufficient tissue thickness for graft procurement, while the palatal mucosa overlying the molar region is relatively thin and fragile. (2) The anterior location is more accessible surgically, as limited mouth opening restricts operative access to the posterior palate (Figure 3C).

### 3.2 Clinical assessment

**FIGURE 4:**
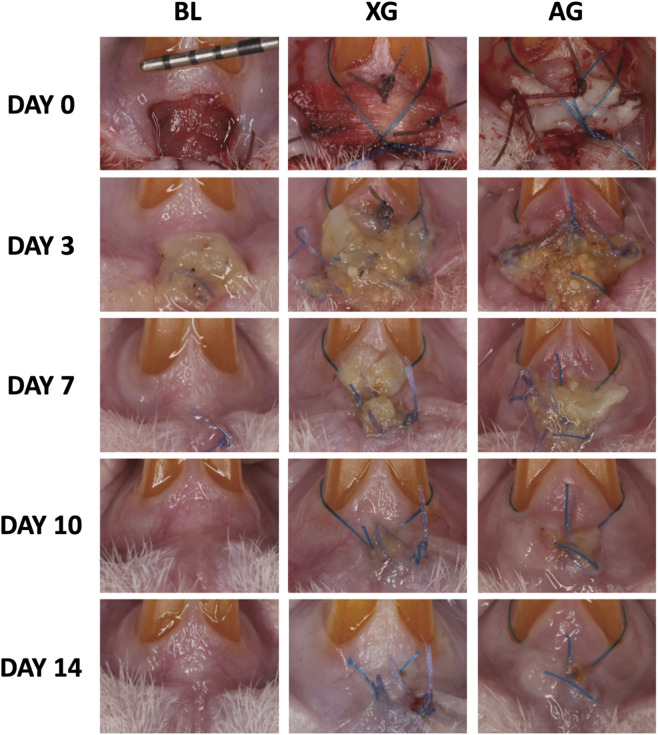
Sequential clinical photographs showing the healing of standardized maxillary anterior soft tissue defects in three groups: blank control (no graft), xenograft, and autograft. Images were taken on Day 0 (immediately after surgery), Day 3, Day 7, Day 10, and Day 14 post-operation.

Throughout the experimental period, all rats recovered uneventfully, with no incidence of bleeding, infection, or significant weight loss. On day 3 postoperatively, the defects were covered with abundant inflammatory exudate. In the AG group, the grafts were completely encapsulated, while in the XG group, the xenogenic material was partially exposed to exudate. By day 7, the wound area had markedly decreased. The AG group showed clear tissue integration at the margins of the graft, with a small amount of necrotic tissue on the surface, and remnants of the xenogenic material were still visible in the XG group. At day 10, the AG group exhibited well-integrated grafts that slightly protruded above the surrounding soft tissue, whereas the XG group showed further reduction in wound size but incomplete closure. By day 14, the AG group presented a whitish, scar-like appearance at the healed site, while complete wound closure was achieved in the XG group. The healing patterns in both groups were consistent with clinical observations in human cases (Figure 4).

### 3.3 Histologic analysis of healing process

**FIGURE 5:**
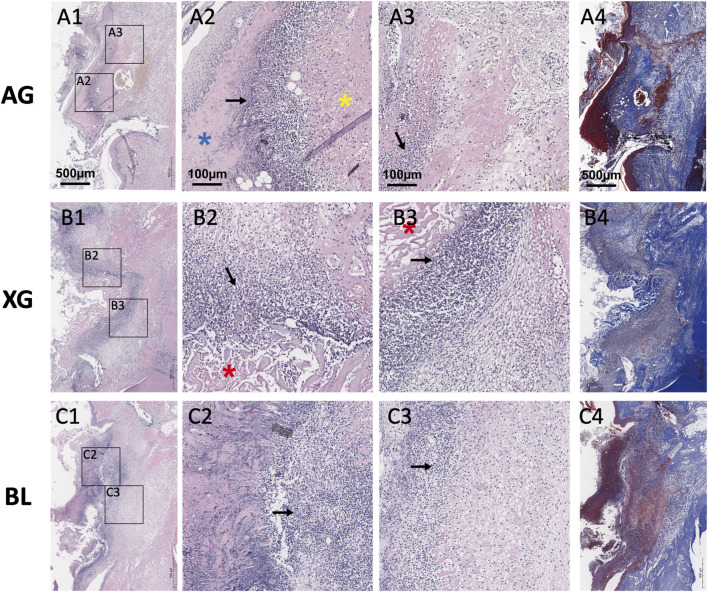
Representative H&E staining (**A1-A3, B1-B3, C1-C3**) and Masson’s trichrome staining (**A4, B4, C4**) images of the blank (BL), xenograft (XG), and autograft (AG) groups on day 3 after surgery. Both AG and XG were securely fixed to the recipient site, with a pronounced inflammatory cell infiltration zone. Black arrows indicate the inflammatory cell infiltration zone. (Blue and yellow asterisks indicate the outer and inner regions of the autograft, respectively. Red asterisks indicate the xenograft.

On day 3 postoperatively, both AG and XG samples revealed that the grafts were securely fixed to the recipient site, with a distinct boundary between graft and host tissue. In the AG group, an intact epithelial layer of the grafts could be observed. A fine fibrous network was observed at the interface, with no significant inflammatory cell infiltration. Within the autografts, densely packed mature collagen fibers were identified. A pronounced inflammatory cell infiltration zone, primarily composed of neutrophils, was located near the soft tissue contour, partitioning the graft into inner and outer regions, with fewer cells in the outer area. In the XG group, incompletely degraded collagen matrix was observed on the surface, with a clear inflammatory infiltration zone on the inner side, where cells migrated into the material pores and adhered to collagen fragments, and a fine fibrous network was observed inside (Figure 5).

**FIGURE 6:**
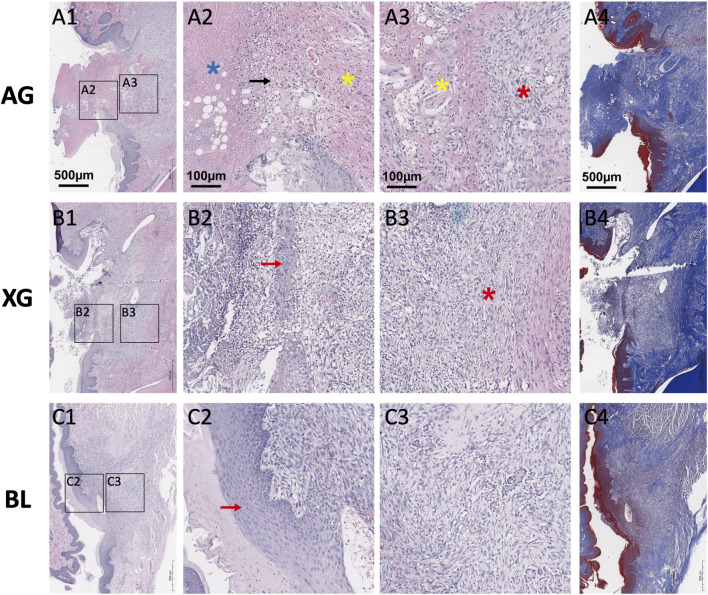
Representative H&E staining (**A1-A3, B1-B3, C1-C3**) and Masson’s trichrome staining (**A4, B4, C4**) images of the blank (BL), xenograft (XG), and autograft (AG) groups on day 7 after surgery. AG remained distinguishable, with partial loss of the epithelium and migration of epithelial cells from the periphery. XG was largely degraded, and abundant fibroblasts and disorganized collagen were observed. Black arrows indicate the inflammatory cell infiltration zone. Red arrows indicate the newly formed epithelium. Blue and yellow asterisks indicate the outer and inner regions of the autograft, respectively. Red asterisks indicate the fibroblasts and disorganized collagen at the graft-host interface.

On day 7, the AG grafts remained distinguishable, with partial loss of the epithelial layer and migration of epithelial cells from the periphery to the centre. The width and density of the inflammatory infiltration zone were significantly reduced compared to day 3. The outer region was nearly acellular, indicating necrosis, while the inner region showed increased cellularity, suggesting tissue remodelling. Numerous fibroblasts were present at the graft–host interface, and mature collagen was intermingled with newly formed collagen, indicating initial integration of the graft with recipient tissue. In the XG group, a thin layer of epithelial cells had formed on the defect surface, though continuity was not fully restored. The graft material was largely degraded, and abundant fibroblasts and disorganized collagen were observed in the newly formed tissue (Figure 6).

**FIGURE 7:**
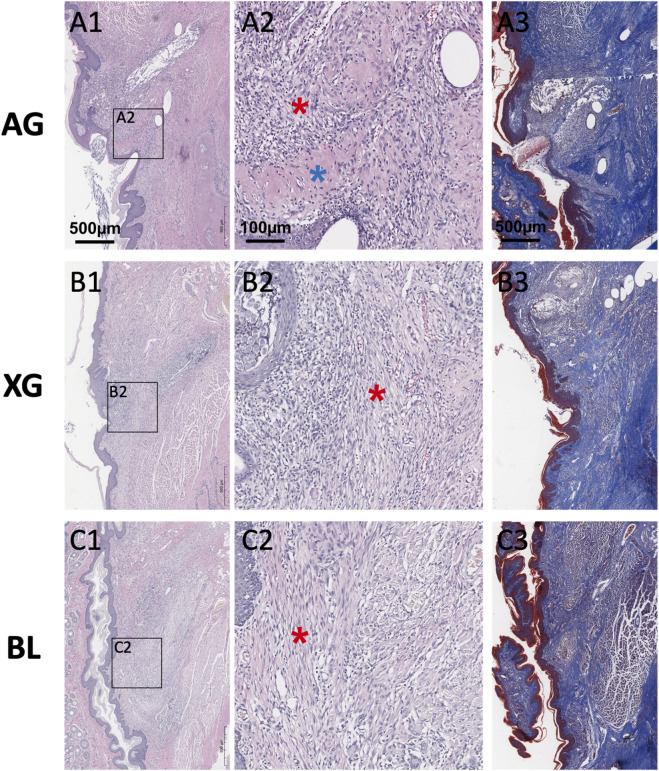
Representative H&E staining (**A1-A2, B1-B2, C1-C2**) and Masson’s trichrome staining (**A3, B3, C3**) images of the blank (BL), xenograft (XG), and autograft (AG) groups on day 14 after surgery. All groups exhibited complete epithelial regeneration. Red asterisks indicate the fibroblasts and disorganized collagen. Blue asterisks indicate the densely arranged collagen.

On day 14, all groups exhibited complete epithelial regeneration. In the AG group, large numbers of fibroblasts and newly formed collagen fibers were present beneath the epithelium, with regions of densely arranged collagen and sparse cellularity. In the XG group, the regenerated tissue in the defect area exhibited abundant fibroblasts and thicker, more organized collagen fibers compared to day 7 (Figure 7).

### 3.4 Vascularization

On day 3, numerous CD31-positive microvessels were observed within the fine fibrous network on the surface of the recipient bed. In the AG group, a small number of microvessels were present on the inner side of the inflammatory infiltration zone, while the outer region was nearly avascular. The XG group exhibited abundant microvessels on the surface of the defect (Supplementary Figure 1). No significant difference in microvessel number was found among the three groups.

On day 7, partial ingrowth of blood vessels into the AG graft was observed, with a marked increase in microvessel density compared to day 3, indicating the onset of revascularization. In the XG group, abundant microvessels were observed in the newly formed tissue. Semi-quantitative analysis revealed a significantly greater number of vessels in the XG group compared to AG and BL, suggesting a pro-angiogenic effect of the xenogenic material (Supplementary Figure 2).

**FIGURE 8:**
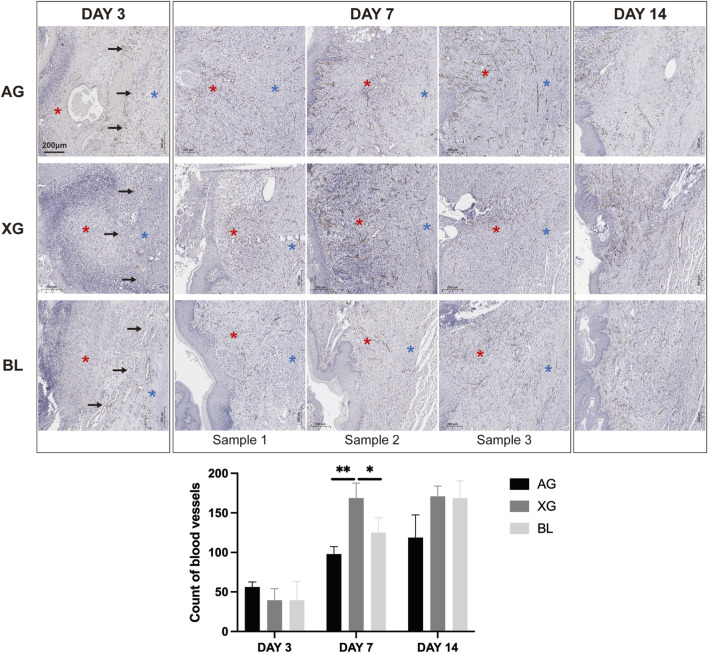
Representative immunohistochemical staining sections and semi-quantitative analysis of CD31 at day 3, 7 and 14 after surgery. Compared to day 3, microvessel density markedly increased on day 7, with significantly more vessels observed in the XG group. Black arrows indicate the surface of the defect. Red asterisks indicate the grafts or regenerative tissue. Blue asterisks indicate recipient bed. Black asterisks indicate significant difference.

On day 14, abundant microvessels were noted in the remodeled autograft, while vessel density was lower in regions with densely arranged collagen. In both the XG and BL groups, numerous microvessels persisted in the regenerated tissue, comparable to those observed at day 7, with no significant difference between the groups (Supplementary Figure 3; Figure 8).

### 3.5 Immune response

**FIGURE 9:**
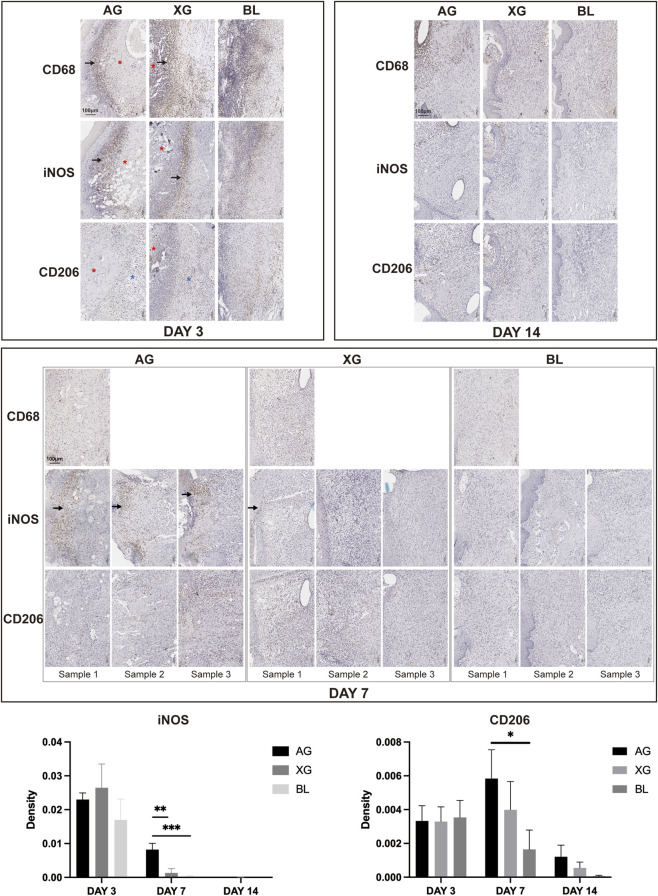
Dynamic changes in macrophage infiltration and polarization during the healing process across autograft (AG), xenograft (XG), and blank (BL) groups. The expression of iNOS gradually decreased from day 3 to day 14 in AG and XG group, while CD206 increased initially and then decreased. Black arrows indicate the inflammatory infiltration zone. Red asterisks indicate the grafts or regenerative tissue. Blue asterisks indicate recipient bed. Black asterisks indicate significant difference.

On day 3, the AG group exhibited dense accumulation of CD68-positive macrophages within the inflammatory infiltration zone, with scattered expression in the inner region. In the XG group, abundant CD68-positive cells were observed in both the infiltration zone and the newly formed tissue. The M1 macrophage marker iNOS was strongly expressed in the infiltration zones of all groups, while the M2 marker CD206 was mainly detected in the newly formed fibrous network at the recipient surface (Supplementary Figures 4, 6). No differences were observed in both the distribution patterns or expression levels of iNOS and CD206 among the three groups (Figure 9).

On day 7, CD68-positive macrophages in the AG group were mainly distributed within the infiltration zone, inside the graft, and at the recipient surface, with lower density compared to day 3. iNOS expression was reduced, while CD206 expression increased in the graft and at the graft-host junction. In the XG group, both CD68 and CD206 expression increased in the newly formed tissue compared to day 3, with scattered iNOS expression observed at the defect surface. Minimal expression of CD68, iNOS, and CD206 was detected in the BL group. Semi-quantitative analysis showed significantly higher iNOS and CD206 expression in the AG group compared to the BL group, while no significant difference was observed between the XG and BL groups (Figure 9).

On day 14, scattered CD68-positive macrophages were detected in the newly formed tissue in both the AG and XG groups but were nearly undetectable in the BL group. The AG and XG groups exhibited similar macrophages distribution pattern. iNOS-positive macrophages were mainly observed around the suture sites, whereas CD206-positive macrophages were distributed within the regenerated tissue (Supplementary Figures 5, 7; Figure 9).

## 4 Discussion

Regeneration and repair of periodontal soft tissue defects remain a central challenge in clinical practice and basic research, especially in the fields of biomaterials and tissue engineering (Liu et al., 2025; Rahimnejad et al., 2024). Although numerous novel soft tissue substitutes have been developed in recent years, the lack of suitable small animal models has limited in-depth investigations into their *in vivo* biological effects and mechanisms (Ríos-Osorio et al., 2025; Chen et al., 2023). In this study, we utilized the rat as an experimental model, systematically analyzed its oral soft and hard tissue structures, and established a standardized anterior maxillary soft tissue defect model. By employing both autologous grafts and clinically relevant xenogenic materials for defect reconstruction, we observed healing patterns that closely resembled those seen in human clinical samples (Rahimnejad et al., 2024), thereby demonstrating the feasibility and translational relevance of this model.

Rat hard palate and buccal mucosal defect models have been widely used in studies of oral wound healing (Toma et al., 2021). However, these models cannot accurately recapitulate the process of periodontal soft tissue regeneration. Compared with the posterior labial region, the anterior site offers a wider surgical field, superior accessibility, and reduced influence from masticatory and licking forces, thereby facilitating both postoperative monitoring and sample collection. Accordingly, to determine the optimal site for periodontal soft tissue defect creation, we compared the anatomical characteristics of maxillary and mandibular anterior labial regions in rats. Though previous studies have reported the use of rat mandibular anterior region, maxillary anterior labial region was ultimately selected as the optimal site in the present study (Paula et al., 2021; Li et al., 2025). The decision was primarily based on the anatomical characteristics of the mandibular anterior mucosa, which is relatively thin and extensively overlies the tooth surface. Such features may increase surgical difficulty, compromise local blood supply, elevate the risk of postoperative gingival recession, and reduce graft survival and revascularization.

Regarding the donor site, both previous research and our histological evidence indicate that rat palatal mucosa closely resembles human palatal tissue, making it highly representative and clinically relevant (Sa et al., 2016). We selected the anterior palate primarily because it offers superior accessibility and visibility during surgery, which is particularly important given the limited oral opening of rats and the need for procedural standardization to minimize inter-animal variability (Xu et al., 2023). Although anatomically comparable, the mid- and posterior-palatal regions are less accessible and more technically challenging due to anatomical curvature and proximity to the molars, complicating both graft harvesting and wound management. Moreover, the mucosa in these regions is relatively thin, and harvesting a 5-mm-wide tissue graft can easily injure the bilateral submucosal vascular bundles, increasing the risk of bleeding and even mortality. In contrast, although abundant vascular bundles are presented in the anterior palatal submucosa, meticulous partial-thickness dissection that preserves the periosteum and deep connective tissue can effectively minimize the risk of bleeding. In this study, no hemorrhage- or asphyxia-related complications were observed, supporting the feasibility of this region as a donor site.

The reproducibility and reliability of an animal model largely depend on the standardization of surgical protocols and meticulous attention to technical details. In this study, we selected 14-week-old, 450–550 g male SD rats, ensuring sufficient tissue area for standardized defect creation as well as optimal regenerative capacity. The distance from the incision to the gingival margin was strictly controlled (no less than 1 mm), and sharp dissection was employed to prevent exposure of the tooth or bone surface, thereby minimizing the risk of postoperative gingival margin necrosis and recession. Furthermore, considering the potential confounding effects of muscle contraction during wound healing on outcome evaluation, mattress sutures were used to secure the apical mucosa to the periosteum, thereby minimizing this interference. Taking the above factors into account, the defect size was limited to 4 × 2 mm. These technical refinements ensured the stability and comparability of the model, enabling systematic evaluation of different materials at multiple time points.

This model is particularly suitable for the early-stage evaluation of the histological behavior of autografts and soft tissue substitutes in periodontal soft tissue defect repair. Through various staining techniques, we were able to comprehensively assess critical biological processes such as material degradation, collagen deposition and remodeling, angiogenesis, and immune cell infiltration. In particular, IHC staining for macrophage markers revealed a typical transition from M1 to M2 polarization, which plays a critical role in soft tissue regeneration. M1-like macrophages are primarily associated with the inflammatory response by producing cytokines that contribute to pathogen clearance. However, excessive or sustained inflammation may impair the progression of wound healing. Meanwhile, anti-inflammatory M2-like macrophages are involved in tissue repair by resolving inflammation, activating myofibroblast and promoting angiogenesis, thereby facilitating tissue remodeling (Sekar et al., 2026; Sim et al., 2022). Variations in the expression levels and infiltration patterns of iNOS and CD206 among the groups may reflect distinct immune response as well as different healing patterns and stages of the healing process. Compared to large animal models, rats provide significant advantages in terms of ethical compliance with the 3R principles, cost-effectiveness, and technical manageability, while permitting large sample sizes and detailed multi-time-point analyses (Rinwa et al., 2024). This model’s established genetic background and the availability of molecular tools also facilitate in-depth mechanistic studies, enabling the investigation of gene and signaling pathways involved in soft tissue regeneration. While larger animals may offer closer anatomical similarity to humans, the rat model’s balance of translational value, throughput, and accessibility makes it particularly well-suited for screening and mechanistic research at the preclinical stage (Domínguez-Oliva et al., 2023). Such strengths ensure that findings from rat palatal models can provide a strong scientific foundation for subsequent validation in higher-order animal models and eventual clinical translation.

Nevertheless, this study has certain limitations. First, the untreated defects in the blank group exhibited spontaneous healing and a faster rate of closure than those in the xenograft group, suggesting that the defect does not represent a critical-size defect. Accordingly, this model is primarily intended for mechanistic investigations of graft materials using histological and molecular biological approaches. Notably, this limitation should be taken into consideration when interpreting outcomes based on wound closure rates, as the model is not suitable for definitive efficacy evaluation using closure rate as the primary endpoint. Second, although new tissue formation was observed at 14 days postoperatively, the regenerated tissue had not yet reached full maturity, and thus the model is mainly suited for assessment of early biological responses after graft implantation. Future studies may consider extending the follow-up period or incorporating functional assessments to further enhance the value of this model.

**FIGURE 10:**
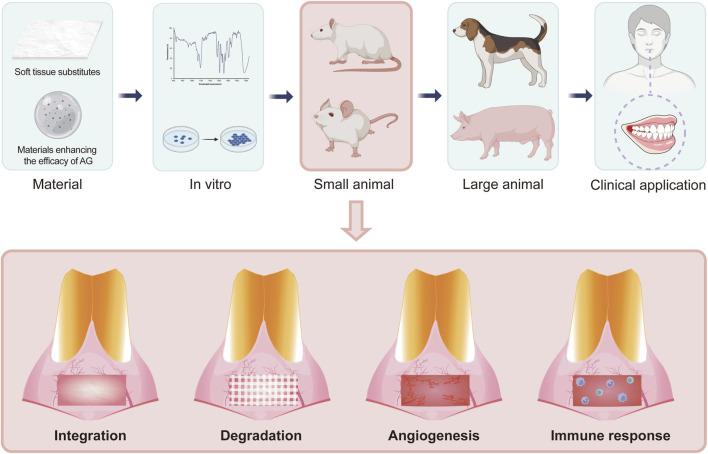
Potential application of this animal model.

The establishment of a standardized rat maxillary anterior periodontal soft tissue defect model holds significant value for the future development of soft tissue substitutes, as well as drugs or materials for enhancing the efficacy of autogenous grafts (Liu et al., 2019; Liu et al., 2018; Liu et al., 2024). By providing a reproducible and anatomically relevant *in vivo* platform, this model enables systematic and quantitative comparison of different biomaterials, which facilitates early-stage screening and optimization prior to large animal or clinical studies. Mucograft® was selected as a representative material for model establishment and verification in this study because its biological effects are supported by substantial preclinical and clinical evidence. It should be noted that the results cannot represent the overall biological effects of all xenografts. The high degree of surgical standardization and consistent anatomical landmarks minimize inter-animal variability, thereby improving the reliability and comparability of experimental results. Importantly, this model allows for dynamic, multi-time-point evaluation of key biological processes, such as integration, degradation, angiogenesis, and immune response, under conditions that closely mimic the clinical environment (Figure 10). Such comprehensive and longitudinal assessments are crucial for elucidating the mechanisms that govern graft performance and for identifying potential bottlenecks in material design, such as insufficient vascularization or adverse host responses. Furthermore, the use of rats, with their well-characterized genetics and amenability to molecular manipulation, opens new avenues for mechanistic studies, gene editing, and high-throughput evaluation of next-generation regenerative materials. Collectively, this model provides a robust *in vivo* platform that bridges the gap between *in vitro* assays and downstream investigations, including large-animal studies and clinical trials, thereby accelerating the development and optimization of advanced soft tissue graft materials for periodontal and oral reconstructive applications.

## 5 Conclusion

We successfully established a standardized, minimally invasive and reproducible rat periodontal soft tissue defect model with a low risk of complication, which allows *in vivo* assessment of soft tissue graft materials. The model supports early-stage screening and optimization prior to large animal and clinical translation, thereby facilitating the development of advanced biomaterials for periodontal and oral reconstructive applications.

## Data Availability

The original contributions presented in the study are included in the article/Supplementary Material, further inquiries can be directed to the corresponding authors.
