# Supplementary material for: A standardized rat model of maxillary anterior periodontal soft tissue defect for the evaluation of soft tissue graft materials
Source: Front Bioeng Biotechnol. 2026 Apr 20;14:1802902. doi: 10.3389/fbioe.2026.1802902 (PMC13136157; doi:10.3389/fbioe.2026.1802902)
Supplement: Supplementary file 2 [file DataSheet2.pdf]

Supplementary Material

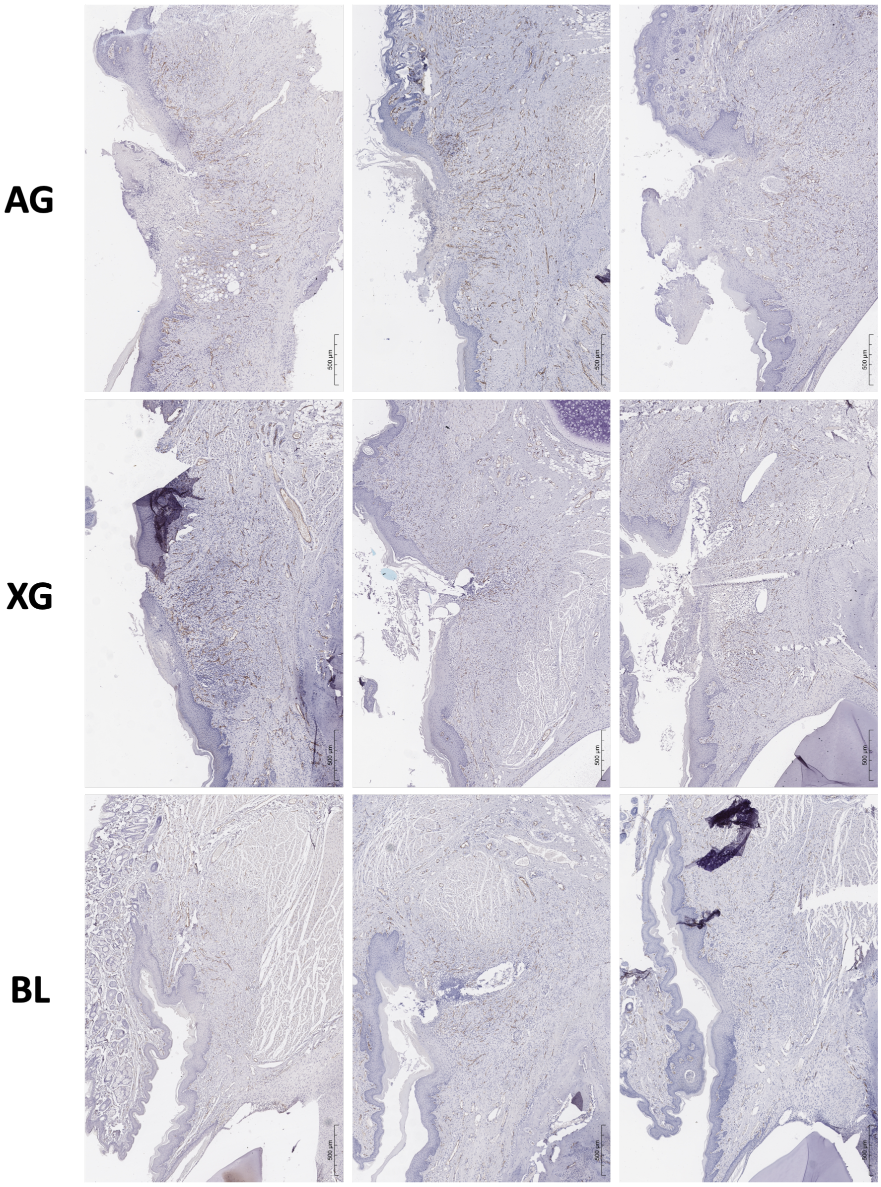

Supplementary Figure 2. Immunohistochemical staining sections of CD31 at day 7
